# Mutagenic Analysis of the C-Terminal Extension of Lsm1

**DOI:** 10.1371/journal.pone.0158876

**Published:** 2016-07-19

**Authors:** Ashis Chowdhury, Swathi Kalurupalle, Sundaresan Tharun

**Affiliations:** Department of Biochemistry, Uniformed Services University of the Health Sciences (USUHS), 4301, Jones Bridge Road, Bethesda, MD, 20814–4799, United States of America; Colorado State University, UNITED STATES

## Abstract

The Sm-like proteins (also known as Lsm proteins) are ubiquitous in nature and exist as hexa or heptameric RNA binding complexes. They are characterized by the presence of the Sm-domain. The Lsm1 through Lsm7 proteins are highly conserved in eukaryotes and they form a hetero-octameric complex together with the protein Pat1. The Lsm1-7-Pat1 complex plays a key role in mRNA decapping and 3’-end protection and therefore is required for normal mRNA decay rates *in vivo*. Lsm1 is a key subunit that is critical for the unique RNA binding properties of this complex. We showed earlier that unlike most Sm-like proteins, Lsm1 uniquely requires both its Sm domain and its C-terminal extension to contribute to the function of the Lsm1-7-Pat1 complex and that the C-terminal segment can associate with the rest of the complex and support the function even in *trans*. The studies presented here identify a set of residues at the very C-terminal end of Lsm1 to be functionally important and suggest that these residues support the function of the Lsm1-7-Pat1 complex by facilitating RNA binding either directly or indirectly.

## Introduction

mRNA turnover is an important control point in gene expression. A major mRNA decay pathway (5’ to 3’ pathway) conserved in all eukaryotes is initiated by deadenylation following which the deadenylated mRNA is decapped by the decapping enzyme and then degraded by the 5’ to 3’ exonuclease, Xrn1 [1,2]. Decapping is a crucial, rate-limiting step in this process and the cytoplasmically localized hetero-octameric Lsm1-7-Pat1 complex is essential for the normal rates of mRNA decapping *in vivo* [1,3].

The Lsm1-7-Pat1 complex is made up of the Pat1 subunit and the seven Sm-like protein subunits, Lsm1 through Lsm7. The Lsm1 through Lsm7 proteins are highly conserved in all eukaryotes and they belong to the large family of Sm-like proteins that is ubiquitous in nature [4–7]. The members of this family (which includes the highly conserved eukaryotic spliceosomal Sm proteins) are typically small proteins characterized by the presence of the Sm domain and they exist in nature as six or seven membered homo or heteromeric complexes that bind RNA and carry out various RNA related functions [5,8,9]. Structural studies have revealed that the quaternary structure of the Lsm1-7 complex is analogous to that of the Sm complex with the seven Lsm proteins arranged in the form of a ring in the order Lsm1-2-3-6-5-7-4 [10,11].

The Lsm1-7-Pat1 complex promotes mRNA decay through the 5’ to 3’ pathway by facilitating mRNA decapping via an unknown mechanism [4–7]. Also, this complex protects mRNA 3’-ends from trimming *in vivo* [12–15]. Consistent with these functions and the fact that decapping is deadenylation dependent in the 5’ to 3’ mRNA decay pathway, purified yeast Lsm1-7-Pat1 complex has RNA binding activity, exhibits a unique binding preference for oligoadenylated RNAs over polyadenylated RNAs and binds at the 3’-ends of RNAs [16]. Further, it preferentially associates at the 3’-ends of deadenylated mRNAs *in vivo* [6,17,18]. The ability of the Lsm1-7-Pat1 complex to bind RNA and to recognize the presence of the 3’-oligo(A) tail is essential for normal rates of mRNA decay *in vivo* and the residues in the Sm domain of Lsm1 are important determinants of such ability [19]. Interestingly, while in the case of most Sm-like proteins the Sm domain is sufficient for their RNA binding and oligomerization functions, we showed that Lsm1 is unique in requiring both its Sm domain and its ~57 residues long C-terminal extension for its ability to support the RNA binding activity of the Lsm1-7-Pat1 complex [20]. *lsm1* mutants expressing truncated versions of Lsm1 lacking part or whole of the C-terminal extension exhibit a strong defect in mRNA decay *in vivo* although they are able to assemble the Lsm1-7-Pat1 complex. The mutant Lsm1-7-Pat1 complexes purified from such mutants are severely impaired in their RNA binding activity although they retain the ability to recognize the oligo(A) tail of the RNA [14,20].

The manner in which the C-terminal extension of Lsm1 affects the RNA binding activity of the Lsm1-7-Pat1 complex is not known. Structural studies on Lsm1-7 complex have revealed that the C-terminal extension of Lsm1 starts at the proximal face of the ring structure, wraps around the side of the ring as a short α-helix, bends and crosses the entire diameter of the distal face of the ring forming a longer α-helix and ends with the last segment of the C-terminal extension bent over the longer helix [10,11]. As a result, the C-terminal extension of Lsm1 makes contacts with multiple Lsm subunits [10,11]. In the present study we have carried out mutagenic analysis of the C-terminal extension of Lsm1 to reveal the residues that are important for its function. Our results suggest that these residues act by facilitating RNA binding.

## Materials and Methods

Yeast strains used for all the experiments except those shown in S2 Fig are in the yRP841 genetic background [21]. The *lsm1-27* strain used in these experiments, yST514, was made by transforming the *lsm1Δ* strain yRP1365 [6] with pST324 [20]. Similarly, the other *lsm1* mutants used in this study were also made by transforming the *CEN* plasmids expressing the corresponding alleles (Table 1) into yRP1365. Strains used for experiments in S2 Fig are in the genetic background of BMA64 [22]. The *lsm1-27* strain used in these experiments, yST508, was made by transforming the *lsm1Δ* strain yST188 [20] with pST307. pST307 was made by cloning the NotI insert from pST324 into the NotI site of pRS413. *CEN* plasmids expressing various *FLAG-lsm1* alleles were generated by introducing the appropriate mutations into pST17 [16] via QuikChange mutagenesis. C-terminal extension coding regions were then PCR amplified from these *CEN* plasmids and cloned into pESC-URA (Agilent Technologies) vector to generate *2μ* vectors that over express the C-terminal segments of various *lsm1* alleles. The plasmids made in this study are shown in Table 1.

**Table 1:** Plasmids used this study.

Vector: pRS416^a^		Vector: pESC-URA^b^	
Plasmid	Insert	Plasmid	Insert
pST336	*FLAG-lsm1-30*	pST338	6xHis tagged C-terminal extension of *lsm1-16*
pST331	*FLAG-lsm1-32*	pST339	6xHis tagged C-terminal extension of *lsm1-17*
pSR335	*FLAG-lsm1-33*	pST340	6xHis tagged C-terminal extension of *lsm1-18*
pST332	*FLAG-lsm1-34*	pST341	6xHis tagged C-terminal extension of *lsm1-19*
pST262	*FLAG-lsm1-36*	pST342	6xHis tagged C-terminal extension of *lsm1-20*
pST264	*FLAG-lsm1-37*	pST343	6xHis tagged C-terminal extension of *lsm1-21*
pST266	*FLAG-lsm1-38*	pST344	6xHis tagged C-terminal extension of *lsm1-22*
pST467	*FLAG-lsm1-39*	pST345	6xHis tagged C-terminal extension of *lsm1-23*
pST394	*FLAG-lsm1-40*	pST346	6xHis tagged C-terminal extension of *lsm1-24*
pST395	*FLAG-lsm1-41*	pST457	6xHis tagged C-terminal extension of *lsm1-30*
		pST458	6xHis tagged C-terminal extension of *lsm1-32*
		pST459	6xHis tagged C-terminal extension of *lsm1-33*
		pST460	6xHis tagged C-terminal extension of *lsm1-34*
		pST365	6xHis tagged C-terminal extension of *lsm1-39*
		pST399	6xHis tagged C-terminal extension of *lsm1-40*
		pST400	6xHis tagged C-terminal extension of *lsm1-41*

^a^ Sikorski and Hieter, 1989, *Genetics* 122: 19–27.

^**b**^ Agilent Technologies

RNA isolation from yeast cultures, Northern analyses for *MFA2pG* mRNA, western analysis to visualize FLAG-Lsm1 proteins and UV crosslinking assays were carried out as described earlier [14,16,19,20]. Pull down-based RNA binding assays were carried out as described earlier [23] except that radiolabeled RNA was used at a final concentration of 5 nM in the binding reaction. RNA in the pull down fraction was isolated by phenol:choloroform extraction and ethanol precipitation. It was then electrophoresed alongside untreated substrate RNA (10% of the amount used for the assay) on a urea polyacrylamide gel and then subjected to phosphorimaging. The band intensities of pulled down RNA and 10% of the input RNA were compared to determine the percentage of RNA pulled down in each binding reaction. Synthetic 6xHis-tagged peptides were purchased from Peptide 2.0 (Chantilly, VA).

## Results

### Functionally important residues are present in multiple regions of the C-terminal extension of Lsm1

Lsm1 is a 172 residues long polypeptide with a ~75 residues long Sm-domain flanked by ~40 residues long N-terminal extension and ~57 residues long C-terminal extension. Deletion of the C-terminal extension of Lsm1 does not abolish the assembly of the Lsm1-7-Pat1 complex *in vivo* [20]. Clusters of charged residues along the C-terminal extension were changed to Alanines earlier creating the nine *lsm1* alleles, *lsm1-16* through *lsm1-24* [14]. However, none of these mutants exhibited any strong defects in mRNA decay or 3’-end protection although truncation of the C-terminal extension impaired mRNA decay and 3’-end protection in addition to causing temperature sensitivity of growth [14,20]. These observations suggested that smaller lesions in the C-terminal extension can be tolerated easily.

**Fig 1:**
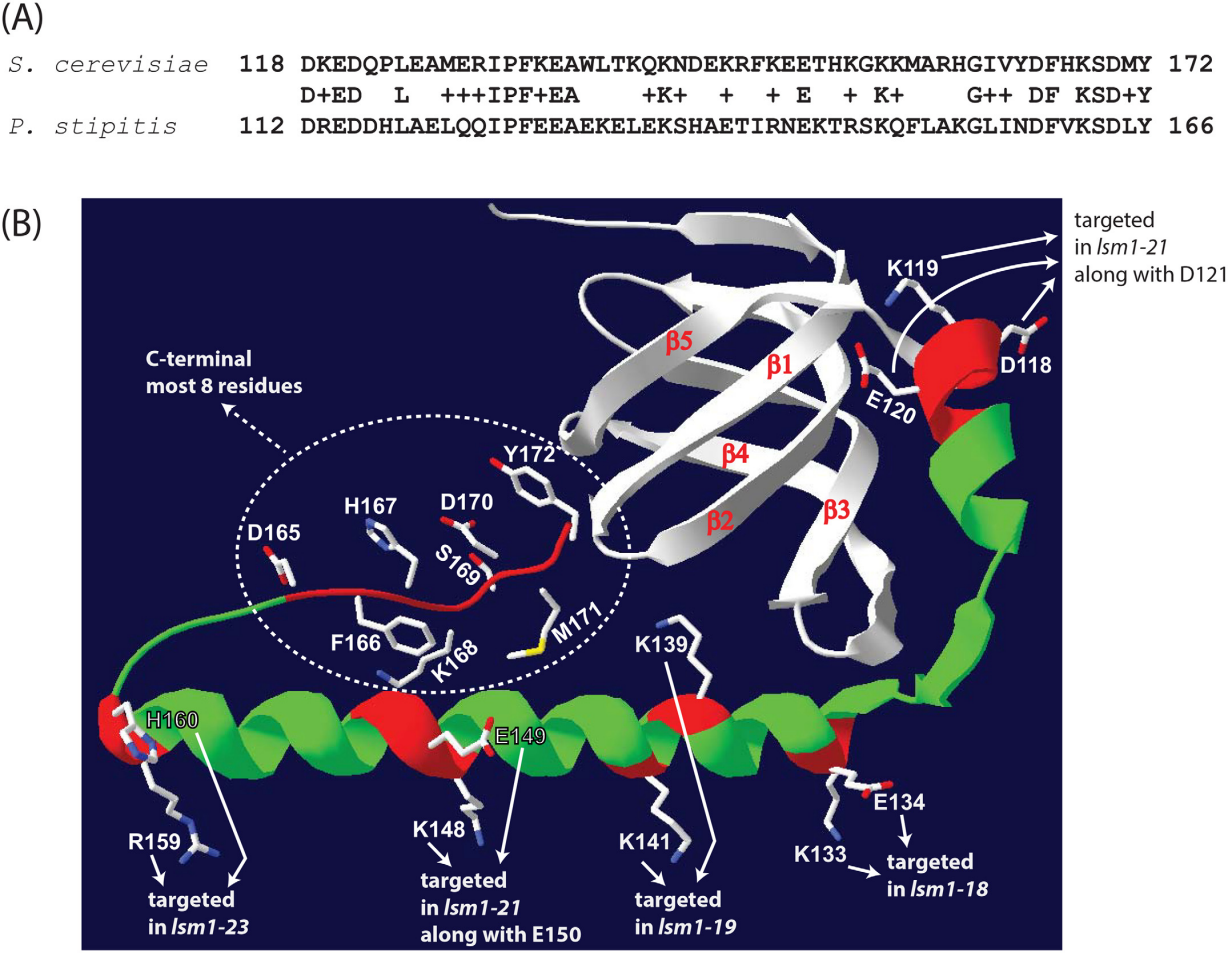
*A*, Alignment of the C-terminal most 55 residues of *S*. *cerevisiae* and *P*. *stipitis* Lsm1 proteins. Numbers of the first and last residues are indicated on the left and right of the sequence. *B*, Three dimensional structure of Lsm1 subunit in the Lsm1-7 complex (PDB ID: 4C92; Sharif and Conti, 2013) is shown as ribbon diagram. Parts of the ribbon corresponding to some of the C-terminal extension residues targeted in the *lsm1* mutants are shown in red. For these residues the orientation of the side chains is also shown. Rest of the C-terminal extension is shown in green. The N-terminal extension and the Sm domain are shown in gray.

BLAST search using the C-terminal most 55 residues of *S*. *cerevisiae* Lsm1 reveals its similarity to the corresponding C-terminal sequences of several fungal Lsm1 orthologs and the C-terminal extension of the *Pichia stipitis* (a.k.a. *Scheffersomyces stipitis*) Lsm1 is among the least similar ones in this list. Nevertheless, the C-terminal extension of *P*. *stipitis* was able to support mRNA decay and 3’-end protection functions *in vivo* in yeast when it was used to replace the C-terminal extension of *S*. *cerevisiae* Lsm1 to generate a chimeric Lsm1 protein [20]. Alignment of the C-terminal most 55 residues of *S*. *cerevisiae* and *P*. *stipitis* Lsm1 proteins reveals that most of their similarity is in the first 18 and the last 12 residues of the sequence (Fig 1A). Therefore in order to introduce larger lesions in the C-terminal extension of *S*. *cerevisiae* Lsm1, we combined mutations targeting these residues. Thus, we generated the allele *lsm1-30* by combining the mutations of *lsm1-16* (D118A, K119A, E120A and D121A) and *lsm1-18* (K133A and E134A) and the allele *lsm1-32* by combining the mutations of *lsm1-16*, *lsm1-18* and *lsm1-24* (D165A, H167A, K168A and D170A). In addition we also generated two more alleles by combining mutations targeting residues in the middle region of the C-terminal extension. Thus, *lsm1-33* was made by combining the mutations of *lsm1-19* (K139A and K141A), and *lsm1-23* (R159A and H160A) and *lsm1-34* was made by combining the mutations of *lsm1-19*, *lsm1-23* and *lsm1-21* (K148A, E149A and E150A). Fig 1B shows the positions of some of these residues in the 3 dimensional structure of the Lsm1 subunit in the Lsm1-7 complex [10] and Table 2 lists the residue changes present in each of the *lsm1* alleles generated in this study.

**Table 2:** Residue changes in the various *lsm1* alleles generated in this study.

Allele	Residue changes	Combines the mutations of alleles*
*lsm1-30*	D118A, K119A, E120A, D121A, K133A, E134A	*lsm1-16* and *lsm1-18*
*lsm1-32*	D118A, K119A, E120A, D121A, K133A, E134A, D165A, H167A, K168A, D170A	*lsm1-16*, *lsm1-18* and *lsm1-24*
*lsm1-33*	K139A, K141A, R159A, H160A	*lsm1-19*, and *lsm1-23*
*lsm1-34*	K139A, K141A, R159A, H160A, K148A, E149A, E150A	*lsm1-19*, *lsm1-21* and *lsm1-23*
*lsm1-36*	Residues 122–136 deleted	
*lsm1-37*	Residues 122–159 deleted	
*lsm1-38*	Residues 138–160 deleted	
*lsm1-39*	K139A, K141A, D165A, H167A, K168A, D170A	*lsm1-19*, and *lsm1-24*
*lsm1-40*	D165L, F166P, H167C, K168I, S169V, D170W, M171D, Y172G	
*lsm1-41*	C-terminal 8 residues (D165 to end) deleted	

* The *lsm1-16* through *lsm1-24* alleles are described in Tharun et al. 2005, *GENETICS*, 170: 33–46.

We expressed each of these alleles (from native promoter using a *CEN* vector) in *lsm1Δ* cells to determine if they are able to suppress the mRNA decay defects of those cells using the *MFA2pG* mRNA reporter. The poly(G) insertion present in the 3’UTR of this reporter mRNA blocks Xrn1 action in *cis* so that a decay intermediate (called poly(G) fragment) accumulates during the decay of this mRNA via the 5’ to 3’ pathway [24]. The steady state accumulation of this fragment is decreased in strains defective in 5’ to 3’ exonucleolysis and/or decapping [14,21]. Therefore, the level of the poly(G) fragment (relative to the full length mRNA) serves as a reasonable approximation of the efficiency of 5’ to 3’ decay. As seen in S1 Fig, the *lsm1-30*, *lsm1-32*, *lsm1-33* and *lsm1-34* mutants exhibited small or no decrease in poly(G) fragment levels compared to wild type cells suggesting that they are not significantly defective in mRNA decay. The *MFA2pG* mRNA reporter is also useful to assess the efficiency of 3’-end protection. Impairment of 3’-end protection function leads to 3’-end trimming of the *MFA2pG* mRNA such that a significant fraction of the poly(G) fragment accumulates in trimmed form (while the trimmed fragment is barely detectable in wild type cells wherein 3’-end protection is unaffected). Therefore, the size of such fraction serves as an approximation of the efficiency of 3’-end protection *in vivo* [14]. We showed earlier that mRNA decay and 3’-end protection functions are very closely linked such that the defects in these two functions correlate very well in all the *lsm1* mutants [14]. Consistent with this, S1 Fig also shows that the amount of poly(G) fragment accumulating in trimmed form in the *lsm1-30*, *lsm1-32*, *lsm1-33* and *lsm1-34* mutants is very little or none while most of the fragment is in trimmed form in *lsm1Δ* cells. Thus, even the larger lesions present in the C-terminal extensions of these combinatorial *lsm1* mutants are not sufficient to cause severe impairment of the Lsm1-7-pat1 complex function.

**Fig 2:**
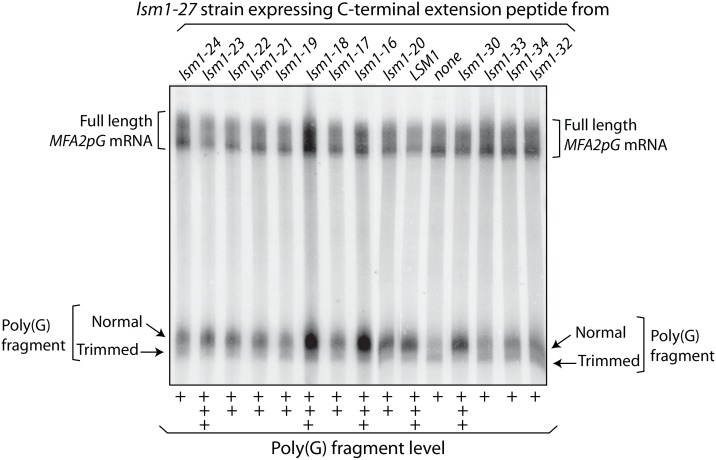
Suppression of the mRNA decay phenotype of the *lsm1-27* mutant (C-terminal truncation mutant of *LSM1*) in *trans* by the various mutant versions of the C-terminal extension peptide, upon over expression. RNA isolated from *lsm1-27* cells expressing wild type or various mutant versions of the C-terminal extension peptide of Lsm1 from multi copy *2μ* vectors were subjected to Northern analysis to reveal the *MFA2pG* mRNA and the poly(G) fragments. The fractional contribution of the poly(G) fragments to the total signal (total = full-length mRNA + trimmed and normal poly(G) fragments) was approximated for each sample via quantitation using the phosphorimager and normalized to the value obtained for *lsm1-27* cells expressing wild type C-terminal extension peptide. Samples with approximate poly(G) fragment levels that are ≥ 80%, 60% to 80% and <60% of the value for cells expressing wild type peptide are marked with +++, ++ and + below the corresponding lanes in the figure.

We had shown earlier that the mRNA decay and 3’-end protection defects of the *lsm1-27* mutant (wherein the entire C-terminal extension of Lsm1 is deleted) can be suppressed by expressing the C-terminal extension (C-terminal most 60 residues, 113 to 172) as a separate polypeptide in that mutant [20]. Thus, the C-terminal extension can support the function of the Lsm1-7-Pat1 complex even when it is not contiguous with the Sm domain of Lsm1 [20]. Since this type of suppression involves the association of the C-terminal extension with the reminder of the Lsm1-7-Pat1 complex in *trans* [20], we reasoned that such suppression may be more sensitive to mutations in the C-terminal extension. Therefore we cloned the C-terminal extensions (C-terminal most 60 residues) from the various *lsm1* alleles discussed above under the Gal promoter in a multi copy *2μ* vector and expressed each of them in *lsm1-27* cells in order to determine their ability to suppress the mRNA decay defect of *lsm1-27* cells using the *MFA2pG* mRNA reporter assays. As shown in Fig 2, we observed that indeed the C-terminal extensions of the combinatorial mutants, *lsm1-32*, *lsm1-33* and *lsm1-34* are weaker than that of the wild type Lsm1 in suppressing these defects *in trans*. The poly(G) fragment level in *lsm1-27* cells expressing these mutant versions of the C-terminal extension peptide is either comparable to or only slightly more than that in *lsm1-27* cells not expressing any C-terminal extension peptide. Further, the fraction of the poly(G) fragment present in trimmed form is significantly higher in the *lsm1-27* cells expressing these mutant peptides compared to *lsm1-27* cells expressing the wild type C-terminal extension peptide. Thus, these results show that the functional defects caused by the lesions in the C-terminal extension that are too weak to be clearly detected when the C-terminal extension is contiguous with the Sm-domain of Lsm1 are accentuated (allowing easy detection) when the phenotypic suppression by the C-terminal extension is studied in *trans*. They also suggest that residues critical for the function are located in multiple parts of the C-terminal extension rather than confined to a small region. The observation that the C-terminal segment of *lsm1-32* allele (mutations of *lsm1-16*, *lsm1-18* and *lsm1-24* combined) was much weaker than that of the *lsm1-30* allele (mutations of *lsm1-16* and *lsm1-18* combined) in its ability to suppress the phenotypes (Fig 2) suggests that the residues targeted in *lsm1-24* allele (D165A, H167A, K168A and D170A) are functionally important. Consistently, the C-terminal segment of *lsm1-24* was also defective in suppressing the mRNA decay defect of the *lsm1-27* cells. Based on this and the observation that the C-terminal extensions of *lsm1-19*, *lsm1-33* and *lsm1-34* are defective in phenotypic suppression, we focused our further analyses on the mutations of the *lsm1-19* and *lsm1-24* alleles. Finally, when this experiment was carried out using yeast strains of a different genetic background, similar results were obtained (S2 Fig).

**Fig 3:**
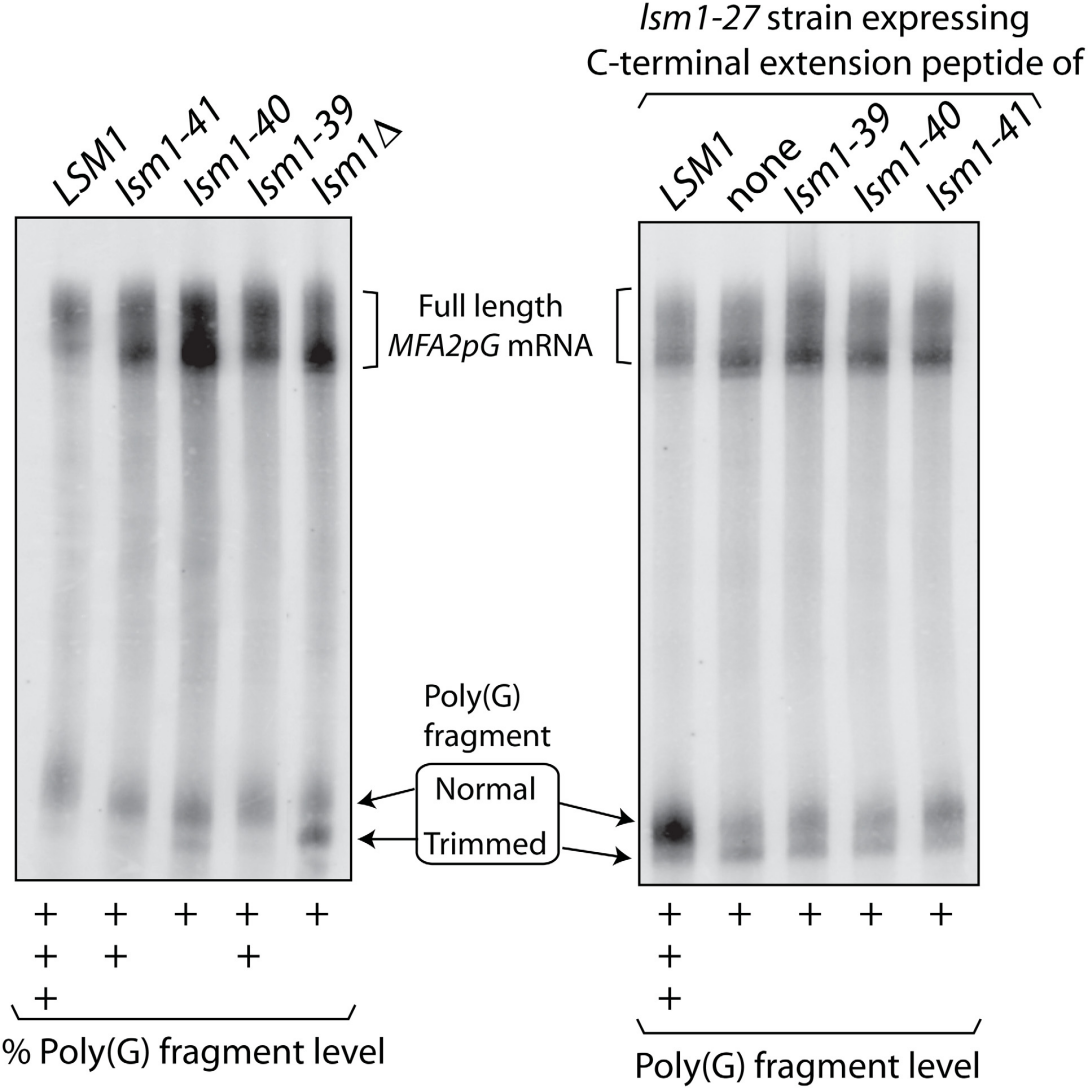
Effect of the C-terminal extension mutations of the *lsm1-39*, *lsm1-40* and *lsm1-41* alleles on mRNA decay. RNA isolated from *lsm1Δ* cells expressing wild type or various mutant alleles of *LSM1* (left panel) or from *lsm1-27* cells expressing wild type or various mutant versions of the C-terminal extension peptide of Lsm1 from multi copy 2μ vectors (right panel) were subjected to Northern analysis to reveal the *MFA2pG* mRNA and the poly(G) fragment. Poly(G) fragment levels were approximated and presented as described in the legend for Fig 2.

**Fig 4:**
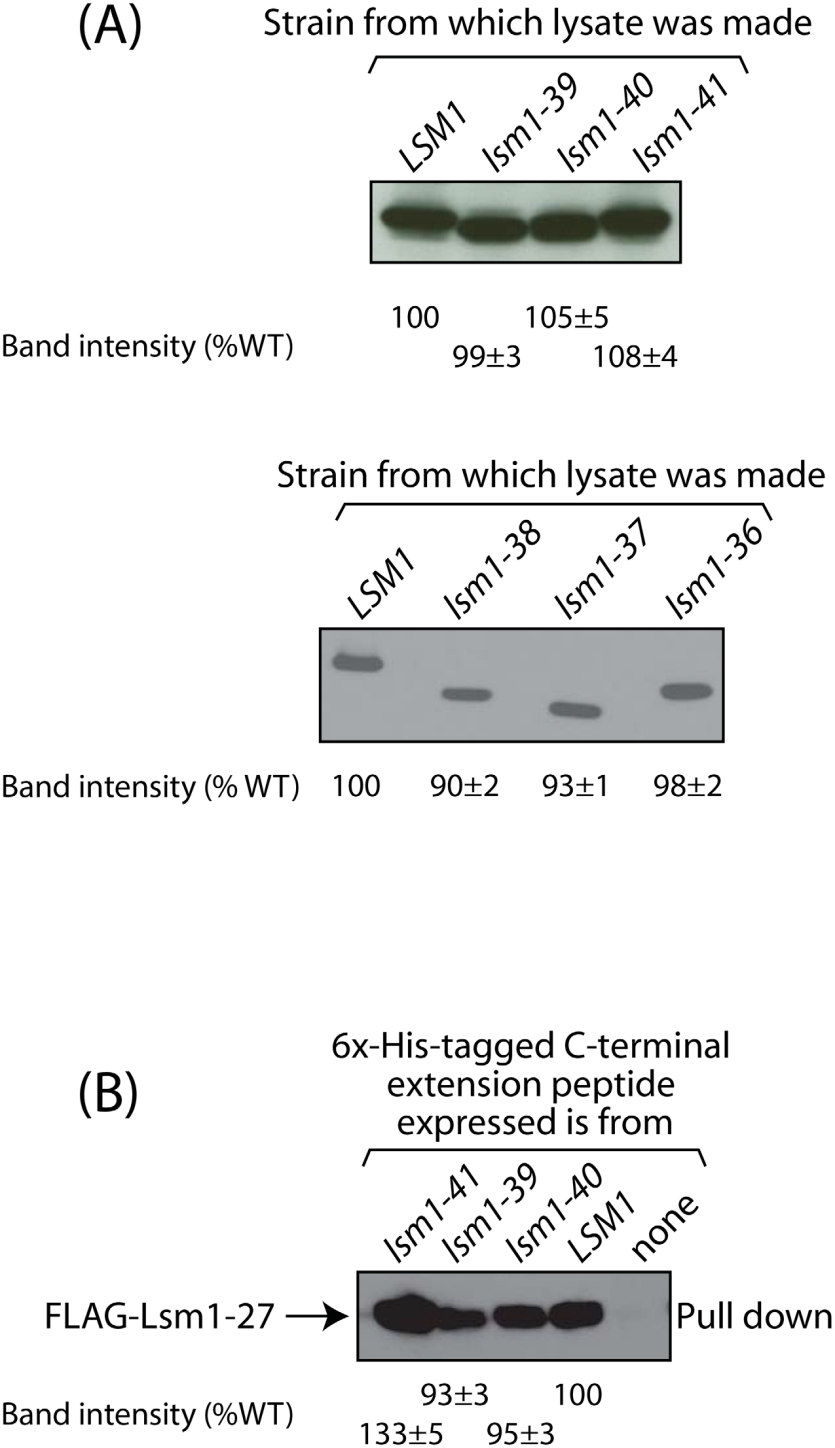
*A*, In *lsm1-36* through *lsm1-41* mutants the corresponding mutant Lsm1 proteins accumulate at levels comparable to that of wild type Lsm1 in wild type cells. *LSM1* and *lsm1-36* through *lsm1-41* cells were grown to log phase and lysates were made from the cells. Equivalent amounts of lysates of *LSM1* and *lsm1-39* through *lsm1-41* cells (upper panel) or *LSM1* and *lsm1-36* through *lsm1-38* cells (lower panel) were subjected to western analysis using anti-FLAG antibodies. *B*, Co-precipitation of FLAG-Lsm1-27 with the C-terminal extension peptide is not significantly impaired by the *lsm1-39*, *lsm1-40* or *lsm1-41* mutations in the peptide. Proteins pulled down from the lysates of *FLAG-lsm1-27* cells over expressing His-tagged C-terminal extension peptides that are wild type or are carrying the *lsm1-39*, *lsm1-40* or *lsm1-41* mutations were subjected to Western analysis using anti-FLAG antibodies. Band intensities quantitated using ImageJ software and normalized to the wild type value are given directly below the lanes of the western blots both in panels *A* and *B*.

The residues changed to Alanine in *lsm1-24* are among those at the very C-terminal end of Lsm1 (D165, H167, K168 and D170) suggesting that this region is important for the function of the C-terminal extension. Therefore, the C-terminal most 8 residues of Lsm1, DFHKSDMY, were either deleted or subjected to most non-conservative substitutions (to LPCIVWDG, as per the Blosum62 matrix) to generate the *lsm1-41* and *lsm1-40* alleles respectively. Additionally, we also combined the mutations of *lsm1-19* and *lsm1-24* to form the *lsm1-39* allele. We then expressed (from native promoter using a *CEN* vector) each of the *lsm1-39*, *lsm1-40* and *lsm1-41* alleles in *lsm1Δ* cells and evaluated their ability to support mRNA decay *in vivo* using the *MFA2pG* mRNA reporter assay. As seen in Fig 3 (left panel), a moderate (*lsm1-39* and *lsm1-41*) to strong (*lsm1-40*) defect in mRNA decay is evident from the decreased accumulation of the poly(G) fragment in these mutants. Thus, the C-terminal extension mutations of these alleles (*lsm1-39*, *lsm1-40* and *lsm1-41*) lead to an mRNA decay defect that is strong enough to be detected even when the C-terminal extension is contiguous with the rest of Lsm1. Western analysis of lysates prepared from the *lsm1-39*, *lsm1-40* and *lsm1-41* mutant cells revealed that these mutants are able to accumulate the corresponding mutant Lsm1 proteins at levels comparable to that of wild type Lsm1 in wild type cells (Fig 4A, upper panel) suggesting that the phenotype of these mutants is not due to their inability to express/accumulate the mutant Lsm1 proteins. In order to determine the effect of these lesions on the ability of the C-terminal extension to function in *trans*, we also cloned the C-terminal extensions of *lsm1-39*, *lsm1-40* and *lsm1-41* alleles (C-terminal most 60 residues in the case of *lsm1-39* and *lsm1-40* and C-terminal most 52 residues in the case of *lsm1-41*) under the Gal promoter in a *2μ* vector and expressed them in the *lsm1-27* mutant to determine their ability to suppress the mRNA decay phenotype of that mutant in *trans*. Fig 3 (right panel) shows that all the three mutant peptides are clearly defective in their ability to suppress the mRNA decay defect of the *lsm1-27* mutant as evident from the decreased poly(G) fragment accumulation in *lsm1-27* cells expressing these mutant C-terminal extension peptides compared to the *lsm1-27* cells expressing the wild type peptide. Further, a significantly higher fraction of the poly(G) fragment accumulated in trimmed form in these cells compared to the cells expressing the wild type peptide. Similar results were obtained when these studies were carried out using yeast strains of a different genetic background (S2 Fig). These observations support the idea that the residues at the C-terminal end of Lsm1 are indeed important for the function and such an idea is also consistent with the observation that C-terminal tagging of Lsm1 results in mRNA decay and 3’-end protection defects [14].

In order to determine if the residues at the C-terminal end of Lsm1 are sufficient to support the function of the C-terminal extension of Lsm1, we also created three more *lsm1* alleles (*lsm1-36*, *lsm1-37* and *lsm1-38*) that carry deletions in the middle and N-terminal portions of the C-terminal extension while retaining the C-terminal most 12 residues. The *lsm1-36*, *lsm1-37* and *lsm1-38* alleles lack residues 122–136, 122–159 and 138–160 respectively (S3 Fig). We expressed (from native promoter using a *CEN* vector) each of these alleles in *lsm1Δ* cells and evaluated their ability to support mRNA decay using the *MFA2pG* mRNA reporter assay. As seen in S3 Fig, a modest defect in mRNA decay is evident from decreased poly(G) fragment levels at least in the case of the *lsm1-36*, and *lsm1-37* mutants. Western analysis of lysates prepared from the *lsm1-36*, *lsm1-37* and *lsm1-38* mutant cells revealed that these mutants are able to accumulate the corresponding mutant Lsm1 proteins (Fig 4A, lower panel) at levels comparable to wild type Lsm1 in wild type cells. These observations suggest that in addition to the residues at the C-terminal end, the residues at the N-terminal portion of the C-terminal extension, especially the residues 122 to 136 are also important for the function of Lsm1. This further supports the idea that functionally important residues are not confined to a particular small region of the C-terminal extension.

### Mutations of *lsm1-39*, *lsm1-40* and *lsm1-41* do not abolish the interaction of the C-terminal extension with Lsm1-27 in *trans*

The results presented above show that the residues in the C-terminal extension of Lsm1 targeted in the alleles, *lsm1-39*, *lsm1-40* and *lsm1-41* are important for the function of the Lsm1-7-Pat1 complex. This could either be because these residues are important for the interaction of the C-terminal extension with the rest of the complex or because they affect the RNA binding activity of the Lsm1-7-Pat1 complex or both. Our earlier studies revealed that the C-terminal extension peptide associates with the mutant Lsm1-7-Pat1 complex containing the C-terminally truncated Lsm1 (Lsm1-27) and enhances its function both *in vivo* and *in vitro* [20]. Importantly, pull down assays showed that when the C-terminal extension is expressed as a separate peptide *in vivo*, it co-precipitates Lsm1-27 but not wild type Lsm1 suggesting that the observed association in *trans* involves native interactions [20]. Consistent with these observations, structural studies have revealed that the residues in the C-terminal extension of Lsm1 interact with other Lsm subunits like Lsm3 and Lsm6 in addition to the other parts of Lsm1 in the Lsm1-7 complex [10,11]. Therefore, to determine if the lesions borne by the alleles, *lsm1-39*, *lsm1-40* and *lsm1-41* impair the interaction of the C-terminal extension with the rest of the Lsm1-7-Pat1 complex, we carried out the pull down assays wherein His-tagged versions of the C-terminal extensions of the *LSM1*, *lsm1-39*, *lsm1-40* and *lsm1-41* alleles were individually expressed in *FLAG-lsm1-27* cells and pulled down using Ni-NTA matrix from the lysates of such cells. Western analysis of the pull down samples revealed that co-precipitation of Lsm1-27 with the mutant versions of the C-terminal extension peptide is not significantly impaired compared to that with the wild type C-terminal extension peptide (Fig 4B). These results suggest that the disruption of the interaction of the C-terminal extension with the rest of the complex is probably not the reason why these mutations impair the function of the C-terminal extension. Further, these results also argue that the inability of the mutant C-terminal extension peptides to suppress the phenotypes *in trans* is not due to their insufficient accumulation/expression compared to the wild type C-terminal extension peptide *in vivo*. These mutations could therefore be affecting the Lsm1-7-Pat1 complex function by impairing the complex’s ability to bind RNA. Additional pull down assays also revealed that Lsm1-27 can be co-precipitated with wild type C-terminal extension peptide from the lysates of *lsm1-27 pat1Δ* cells (S4 Fig) indicating that Pat1 is not required for this interaction. This is consistent with the observation that association of Pat1 with the Lsm1-7 ring is not abolished in the *lsm1-27* mutant (wherein the C-terminal extension of Lsm1 is completely deleted) [20].

### C-terminal extension of Lsm1 is able to bind RNA by itself and the C-terminal most residues are essential for efficient binding

**Fig 5:**
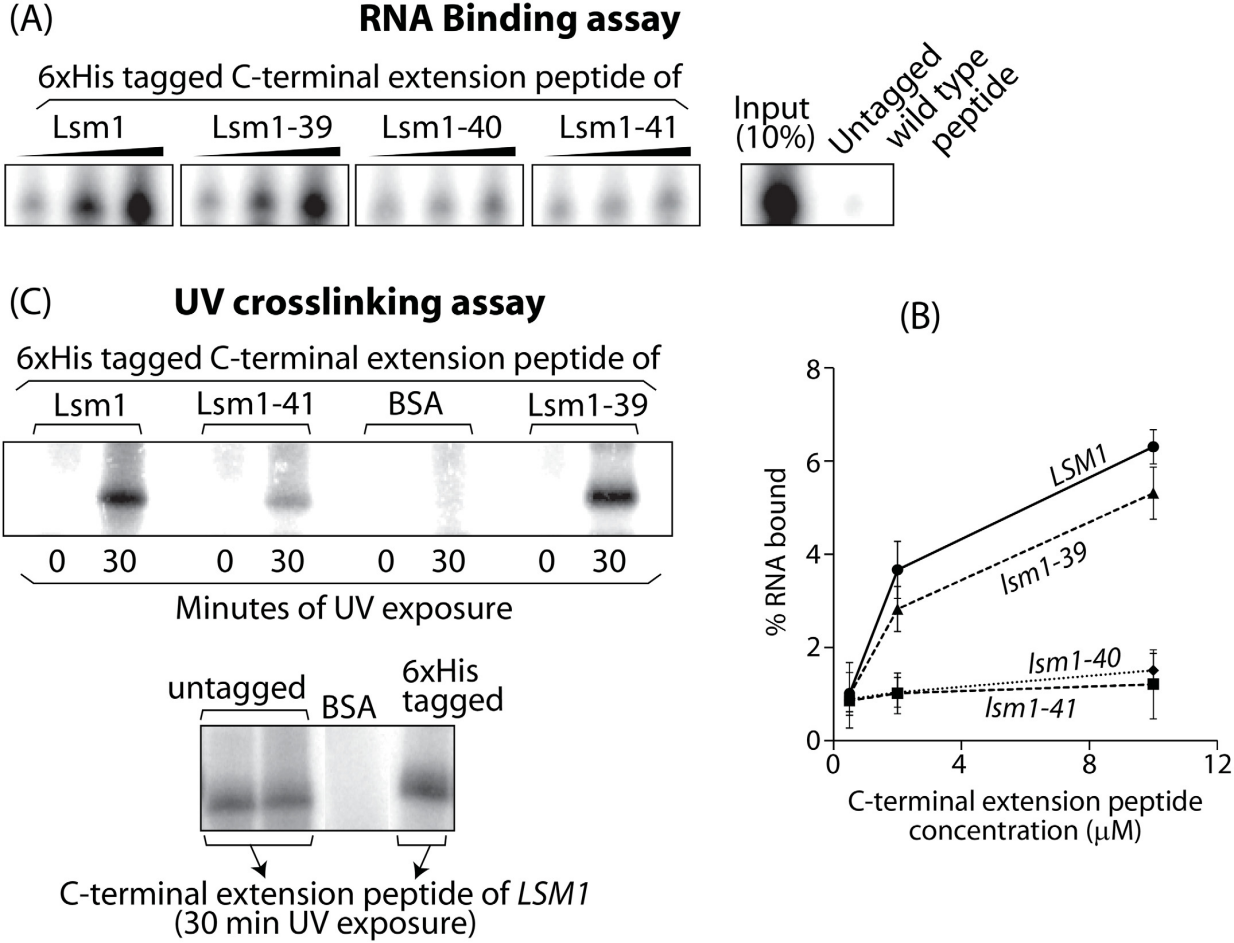
The C-terminal extension of Lsm1 is able to bind RNA by itself and the C-terminal most 8 residues are important for such binding. *A*, Synthetic untagged wild type Lsm1 C-terminal extension peptide (10μM) or increasing concentrations (0.5μM, 2μM and 10μM) of synthetic 6xHis-tagged C-terminal extension peptides corresponding to *LSM1*, *lsm1-39*, *lsm1-40* or *lsm1-41* were incubated with radiolabeled *in vitro* transcript carrying the 3’-most 43 residues of the yeast *MFA2* mRNA and then subjected to pull down using the Ni-NTA matrix. After washing, the co-precipitated RNA was run alongside untreated RNA (10% of total amount used for the binding) and then visualized by denaturing PAGE and phosphorimaging. *B*, A plot of the percentage of RNA bound vs the concentration of the peptide used is shown. Plotted values are mean ± SD from three independent trials. *C*, *Top panel*, bovine serum albumin (BSA) or synthetic C-terminal segment peptides (2 nmols each) corresponding to *LSM1*, *lsm1-39* or *lsm1-41* were incubated with radiolabeled *in vitro* transcript carrying the 3’-most 43 residues of the yeast *PGK1* mRNA, UV crosslinked, treated with ribonuclease and then visualized by SDS-PAGE and phosphorimaging. *Bottom panel*, Similar UV crosslinking analysis carried out with synthetic wild type Lsm1 C-terminal extension peptide (untagged or 6xHis-tagged) or BSA is shown.

Lesions in the C-terminal extension could impair the RNA binding ability of the Lsm1-7-Pat1 complex either because the C-terminal extension residues directly contact RNA and form a separate RNA binding surface or because the interaction of the C-terminal extension with the reminder of the Lsm1-7-Pat1 complex impacts the functionality of the RNA binding surfaces in the rest of the complex. Therefore, in order to determine if the C-terminal extension of Lsm1 can bind RNA directly, we carried out pull down assays using synthetic His-tagged C-terminal extension peptides. Here, the His-tagged peptide is incubated with radiolabeled *in vitro* transcript carrying the 3’-most 43 residues of the yeast *MFA2* mRNA [16] and then pulled down using Nickel matrix. After washing, the RNA retained on the matrix was extracted, subjected to denaturing polyacrylamide gel electrophoresis (PAGE) and visualized by phosphorimaging. These studies revealed that the C-terminal extension of Lsm1 can directly bind RNA. However, the binding affinity is much lower than that of the Lsm1-7-Pat1 complex (*K*_*D*_ ~50 nM [19]) with only ~6% of the RNA bound even when the peptide was used at 10μM concentration in the binding reaction (Fig 5A and 5B). Similar analyses using the C-terminal extension peptides of *lsm1-39*, *lsm1-40* and *lsm1-41* revealed that those of *lsm1-40* and *lsm1-41* (wherein the C-terminal 8 residues are either subjected to nonconservative substitutions or deleted respectively) are clearly impaired in their ability to bind RNA (Fig 5A and 5B). In order to confirm this further, we also carried out UV crosslinking assay. Here, the *LSM1*, *lsm1-39*, and *lsm1-41* C-terminal extension peptides were individually incubated with radiolabeled *in vitro* transcript carrying the 3’-most 43 residues of the yeast *PGK1* mRNA [16] and subjected to UV crosslinking. After ribonuclease treatment, the crosslinked peptides were visualized by SDS-PAGE followed by phosphorimaging. These studies also showed that the C-terminal extension of *lsm1-41* is significantly impaired in getting crosslinked to the RNA (Fig 5C). These results are consistent with the observation that the mutant C-terminal extension peptides bearing the lesions of the *lsm1-40* or *lsm1-41* alleles are defective in their ability to suppress the mRNA decay defect of the *lsm1-27* cells in *trans*. They are also consistent with our earlier studies showing that the C-terminal extension is necessary for the normal RNA binding capacity of the Lsm1-7-Pat1 complex [20]. Altogether, our results suggest that the residues at the very C-terminal end of Lsm1 support the function of the Lsm1-7-Pat1 complex possibly by facilitating RNA interaction.

## Discussion

A unique feature of Lsm1 that distinguishes it from other Sm-like proteins is that not only its Sm domain but also its C-terminal extension is necessary for its function. Our earlier studies showing that C-terminal truncation of Lsm1 causes strong mRNA decay and 3’-end protection defects indicated that the C-terminal extension of Lsm1 is essential for the normal functionality of the Lsm1-7-Pat1 complex [14]. These mutants carried relatively large truncations from the C-terminus of Lsm1 that deleted anywhere from half to the entire C-terminal extension [14]. Further studies revealed that Lsm1-7-Pat1 complex assembly is not affected in these mutants and the functional defects of these mutants are due to impairment of the RNA binding activity of the mutant complexes [20]. Given this, an important question was if the C-terminal extension affects the RNA binding activity of the Lsm1-7-Pat1 complex directly (by providing another RNA binding surface) or indirectly by impacting the conformation of the Lsm1-7-Pat1 complex such that the RNA binding residues in the rest of the complex can function optimally. Further, the residues that are critical for the function of the C-terminal extension were not known.

Our observations suggest that functionally important residues are not confined to a small region but are rather distributed over multiple parts of the C-terminal extension. Although large deletions in the C-terminal extension resulted in significant mRNA decay and 3’-end protection defects, such effects were not observed in the mutants *lsm1-16* through *lsm1-24*, which were generated by individually changing each of the clusters of charged residues along the primary sequence of the C-terminal extension to Alanines [14,20]. Further, even combinatorial mutants wherein more than one cluster of charged residues is targeted exhibited mild or no defects in mRNA decay. Nevertheless, when suppression of the mRNA decay phenotype of the *lsm1-27* mutant was studied in *trans*, C-terminal extensions derived from multiple *lsm1* mutants including *lsm1-24* were found to be defective in such suppression. These results suggested that the residues at the very C-terminal end of the C-terminal extension could be relatively more important, an idea that was supported by the observation that deletion or mutation of the C-terminal most eight residues (*lsm1-41* and *lsm1-40*) can result in clear, though moderate, defect in mRNA decay even when the C-terminal extension is contiguous with the Sm domain of Lsm1 (Fig 3 left panel). Further, the C-terminal extension peptides derived from these *lsm1* mutants are also severely defective in suppressing the mRNA decay defect of the *lsm1-27* mutant in *trans*. Consistently, the C-terminal extension peptides of the *lsm1-40* and *lsm1-41* alleles are clearly impaired in RNA binding *in vitro* compared to the wild type C-terminal extension peptide. In any case, the residues targeted in these alleles are not sufficient for the full functionality of the C-terminal extension because deletions in the N-terminal part of this region (while keeping the C-terminal region intact) also led to moderate mRNA decay defects when the C-terminal extension is contiguous with the Sm domain of Lsm1.

Although the RNA binding activity observed with the C-terminal extension peptide *in vitro* is low, it is not likely to be nonspecific because the mutations of *lsm1-40* and *lsm1-41* which affect mRNA decay *in vivo* also impair the binding *in vitro*. Further, while the C-terminal extension peptide of *lsm1-39* is not significantly impaired in RNA binding compared to the wild type peptide, and the difference in binding between the *lsm1-39* and *lsm1-41* peptides is observed in two different RNA binding assays (pull down based and crosslinking assays).

The basis of the *in vivo* functional defects of the *lsm1-39* mutant is not yet clear. It is possible the effects these mutations have on the interaction of the C-terminal extension peptide with the rest of the complex or RNA are subtle such that they are not detectable by the pull down and RNA binding assays used here. Alternately, these mutations could affect some steps after RNA binding. For example, replacement of the C-terminal extension of yeast Lsm1 with that of human Lsm1 results in strong defects in mRNA decay and 3’-end protection but does not impair complex assembly. Further, the Lsm1-7-Pat1 complex purified from cells expressing such chimeric Lsm1 not only has considerable RNA binding activity but also is able to recognize the presence of oligo(A) tail on the RNA [20].

Structural studies have shown that the residues in the C-terminal segment of Lsm1 interact with not only other residues in Lsm1 but also residues in other subunits of the Lsm1-7 ring [10,11]. However, it is not clear how the C-terminal segment contributes to the RNA binding activity of the Lsm1-7-Pat1 complex. The C-terminal most residues of Lsm1 identified in this study seem to support this complex’s RNA binding activity; however, it is not clear if they do so in a direct or indirect manner.

## Supporting Information

S1 Fig *lsm1* mutants in which more than one cluster of charged residues in the C-terminal extension are changed to alanines are not significantly affected in mRNA decay and 3’-end protection. RNA isolated from *lsm1Δ* cells expressing wild type or various mutant alleles of *LSM1* were subjected to Northern analysis to reveal the *MFA2pG* mRNA and the poly(G) fragment. Poly(G) fragment levels were approximated and presented as described in the legend for Fig 2.
(PDF)

S2 Fig Effect of over expression of the various mutant versions of the C-terminal extension peptide on the mRNA decay phenotype of the *lsm1-27* mutant in BMA64 genetic background. RNA isolated from *lsm1-27* cells expressing wild type or various mutant versions of the C-terminal extension peptide of Lsm1 from multi copy 2μ vectors (right panel) were subjected to Northern analysis to reveal the *MFA2pG* mRNA and the poly(G) fragment. Poly(G) fragment levels were approximated and presented as described in the legend for Fig 2.
(PDF)

S3 Fig Truncation of the N-terminal and middle regions of the C-terminal extension of Lsm1 have moderate effects on mRNA decay. RNA isolated from *lsm1Δ* cells expressing wild type or mutant alleles (*lsm1-36*, *lsm1-37* and *lsm1-38*) of *LSM1* were subjected to Northern analysis to reveal the *MFA2pG* mRNA and the poly(G) fragments. A schematic diagram showing the three deletions studied and the phosphorimage of the Northern blot are shown in the upper and lower panels respectively. Poly(G) fragment levels were approximated and presented as described in the legend for Fig 2.
(PDF)

S4 Fig FLAG-Lsm1-27 interacts with the C-terminal extension peptide in *pat1Δ* cells. Lysates from *FLAG-lsm1-27* and *FLAG-lsm1-27 pat1Δ* strains that do or do not express His-tagged wild type Lsm1 C-terminal extension peptide and proteins pulled down from such lysates using the Ni-NTA matrix were subjected to Western analysis using anti-FLAG antibodies.
(PDF)
