# Supplementary figures and images for: Mutagenic Analysis of the C-Terminal Extension of Lsm1
Source: PLoS One. 2016 Jul 19;11(7):e0158876. doi: 10.1371/journal.pone.0158876 (PMC4951014; doi:10.1371/journal.pone.0158876)

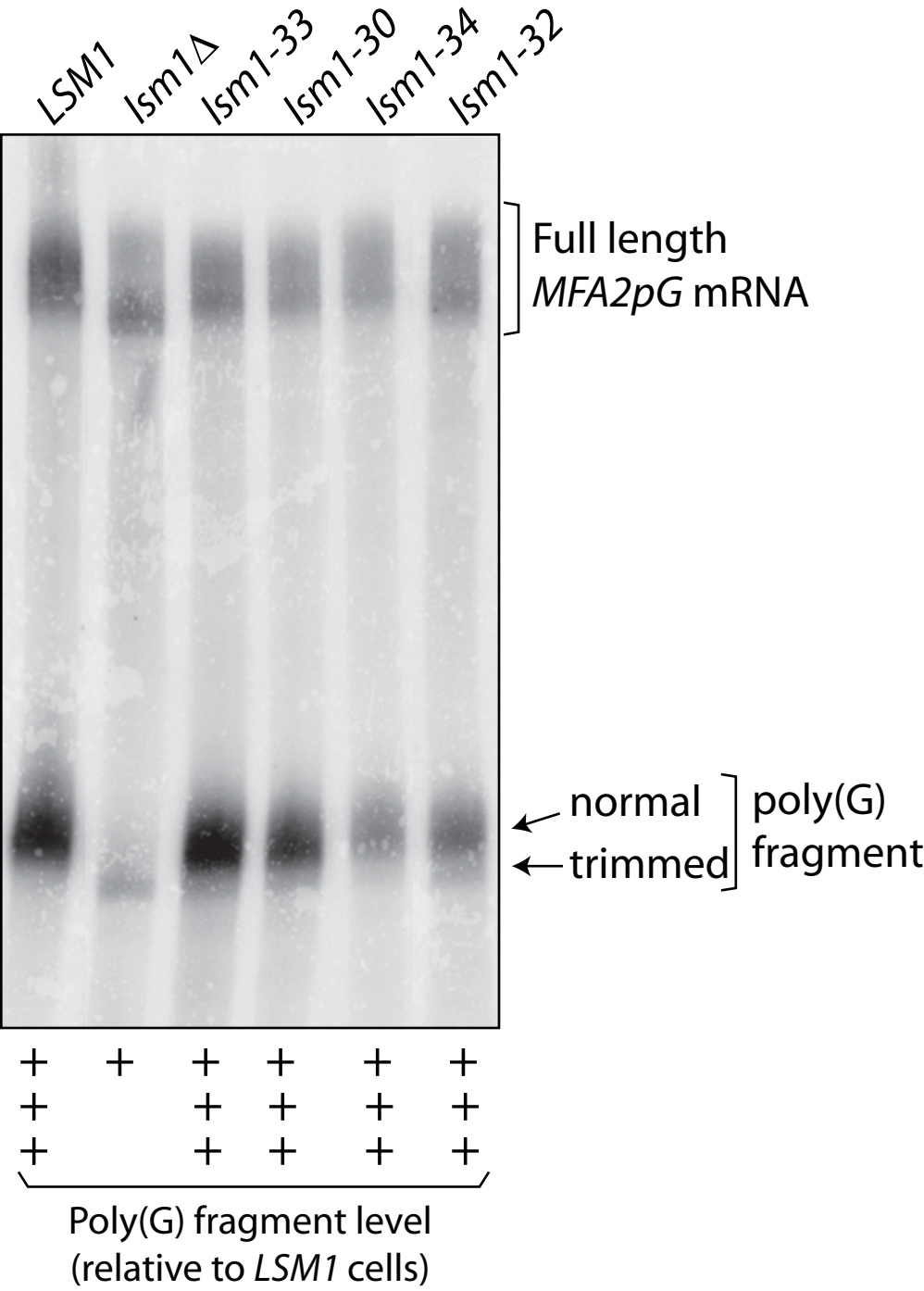

Supplement: S1 Fig — RNA isolated from lsm1Δ cells expressing wild type or various mutant alleles of LSM1 were subjected to Northern analysis to reveal the MFA2pG mRNA and the poly(G) fragment. Poly(G) fragment levels were approximated and presented as described in the legend for Fig 2. (PDF) [file pone.0158876.s001.pdf]

---

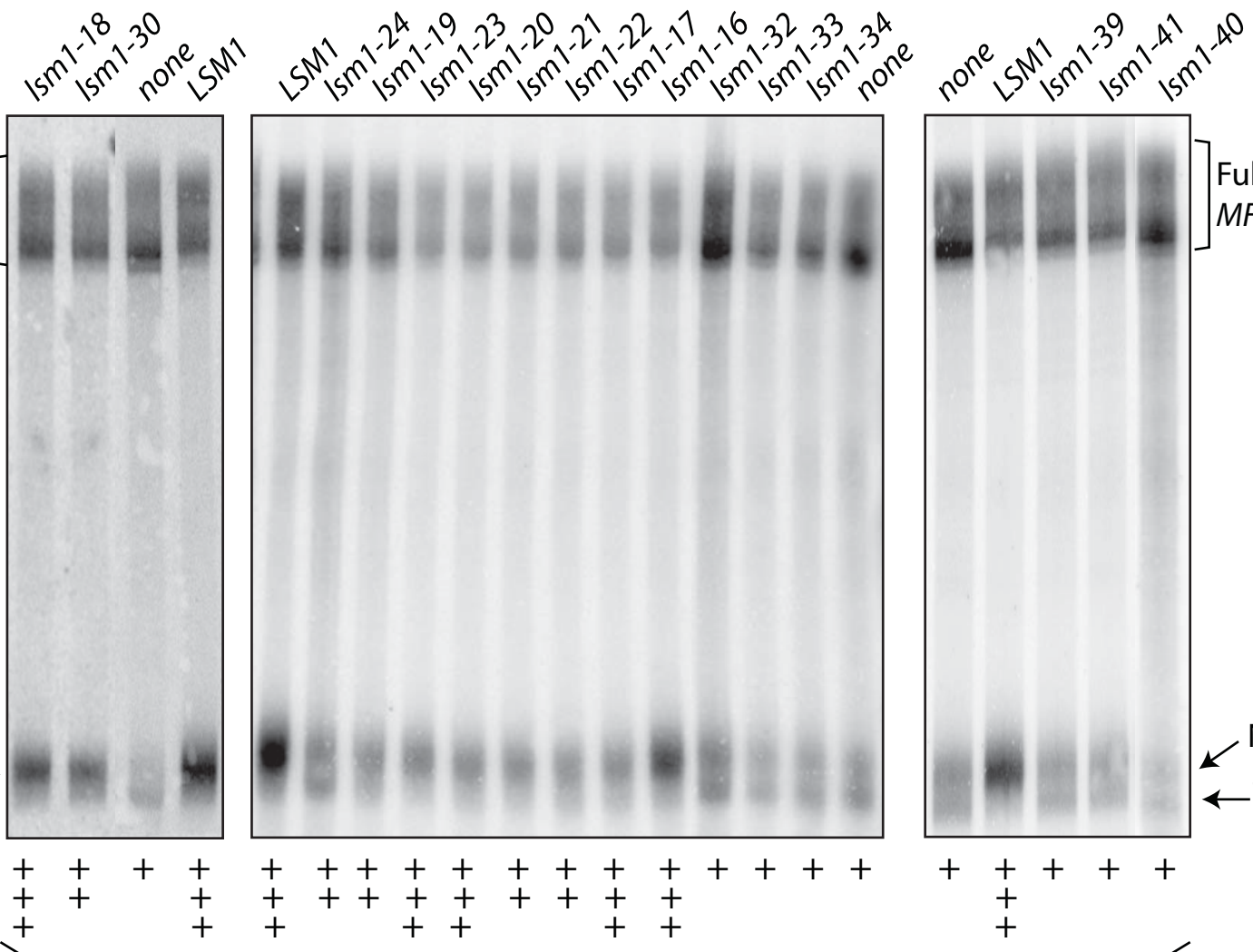

Supplement: S2 Fig — RNA isolated from lsm1-27 cells expressing wild type or various mutant versions of the C-terminal extension peptide of Lsm1 from multi copy 2μ vectors (right panel) were subjected to Northern analysis to reveal the MFA2pG mRNA and the poly(G) fragment. Poly(G) fragment levels were approximated and presented as described in the legend for Fig 2. (PDF) [file pone.0158876.s002.pdf]

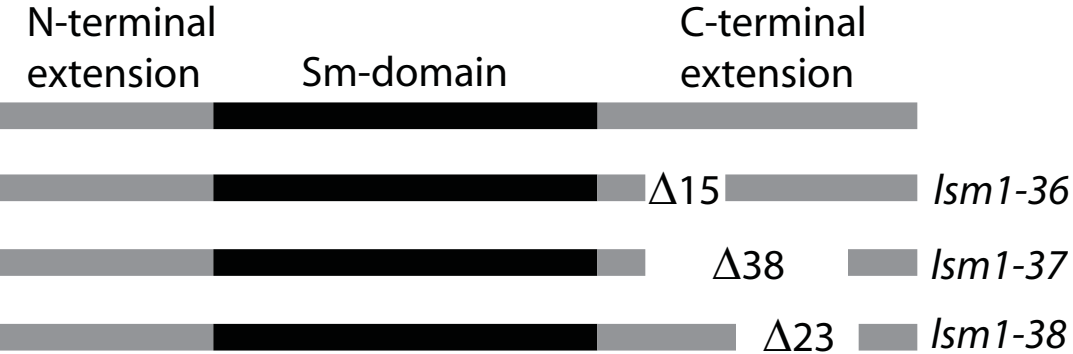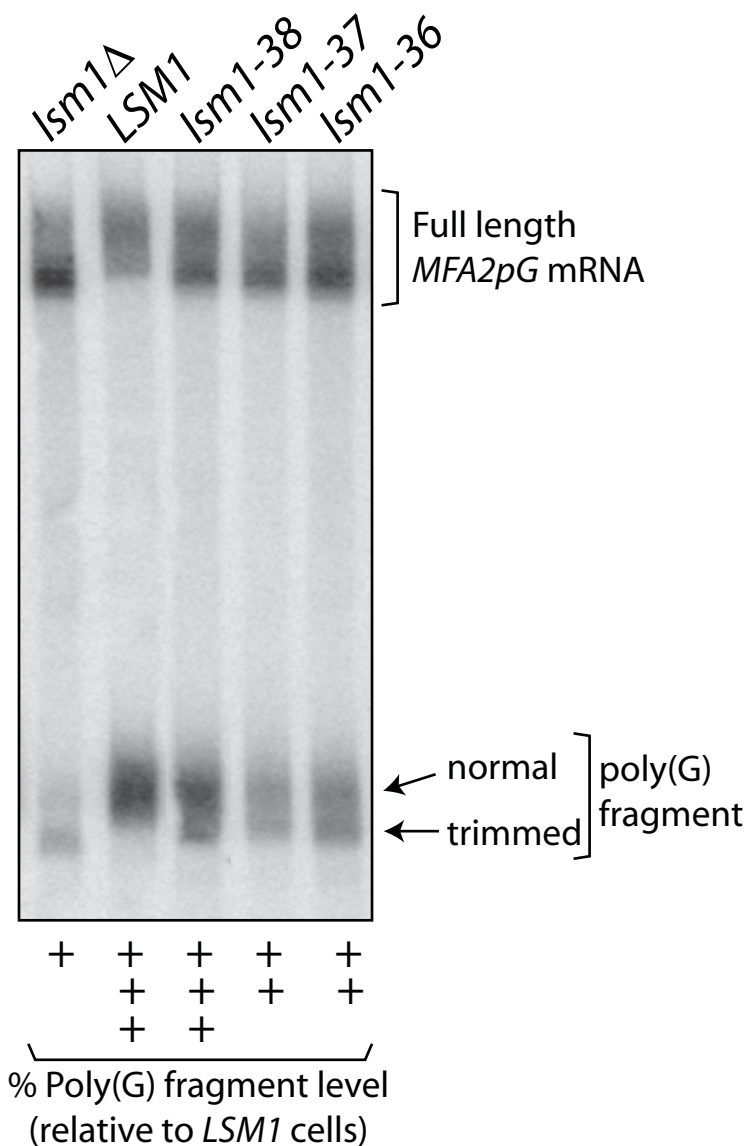

Supplement: S3 Fig — RNA isolated from lsm1Δ cells expressing wild type or mutant alleles (lsm1-36, lsm1-37 and lsm1-38) of LSM1 were subjected to Northern analysis to reveal the MFA2pG mRNA and the poly(G) fragments. A schematic diagram showing the three deletions studied and the phosphorimage of the Northern blot are shown in the upper and lower panels respectively. Poly(G) fragment levels were approximated and presented as described in the legend for Fig 2. (PDF) [file pone.0158876.s003.pdf]

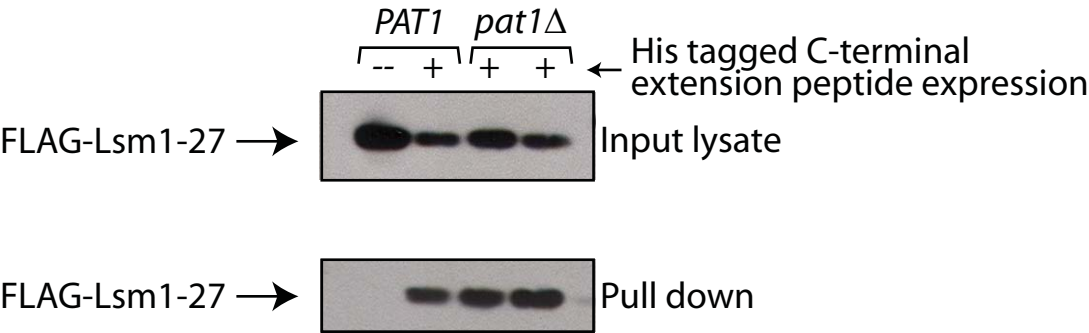

Supplement: S4 Fig — Lysates from FLAG-lsm1-27 and FLAG-lsm1-27 pat1Δ strains that do or do not express His-tagged wild type Lsm1 C-terminal extension peptide and proteins pulled down from such lysates using the Ni-NTA matrix were subjected to Western analysis using anti-FLAG antibodies. (PDF) [file pone.0158876.s004.pdf]
